# In Vitro Study on Green Propolis as a Potential Ingredient of Oral Health Care Products

**DOI:** 10.3390/antibiotics11121764

**Published:** 2022-12-06

**Authors:** Achille Coluccia, Fabienne Matti, Xilei Zhu, Adrian Lussi, Alexandra Stähli, Anton Sculean, Sigrun Eick

**Affiliations:** 1 Department of Periodontology, School of Dental Medicine, University of Bern, 3010 Bern, Switzerland; 2 Preventive and Paediatric Dentistry, School of Dental Medicine, University of Bern, 3010 Bern, Switzerland

**Keywords:** Brazilian green propolis, monocytic cells, biofilm, toothpaste

## Abstract

Propolis is increasingly being discussed as an alternative to commonly used antiseptics. This in vitro study focused on the ethanolic extract of green Brazilian propolis (EEPg) as an additive in an oral health care product. We investigated (i) a potential inflammation-modulation activity of EEPg when a periodontal or Candida biofilm was exposed to monocytic (MONO-MAC-6) cells, (ii) the adhesion of oral pathogens to gingival keratinocytes and (iii) the antimicrobial and antibiofilm effect of different toothpaste formulations. EEPg decreased the levels of interleukin (IL)-1β and increased IL-10 in MONO-MAC cells challenged with a periodontal biofilm. In contact with TIGK cells, EEPg reduced the numbers of adherent *Porphyromonas gingivalis* to 0.5% but did not affect the adhesion of *Candida albicans*. The frequent brushing of a cariogenic biofilm with a toothpaste supplemented with EEPg reduced the surface microhardness loss of enamel specimens. Mixing an experimental erythritol toothpaste with 25 and 50 mg/mL of EEPg confirmed the antibacterial activity of EEPg against oral bacteria and particularly inhibited periodontal biofilm formation. The suggested toothpaste formulations seem to have potential in the prevention of caries, gingivitis and periodontitis and should be evaluated in further in vitro research and in clinical trials.

## 1. Introduction

In recent years, propolis is increasingly being discussed as an alternative to the commonly used antiseptics. Honeybees produce the propolis by mixing plant extracts with beeswax [1]. Propolis consists of up to 300 different components, and it is rich in polyphenols and terpenoids [2]. Propolis has been shown to have antimicrobial activity against bacteria, e.g., *Staphylococcus aureus*, *Pseudomonas aeruginosa*, fungi and certain viruses, and its use is encouraged in infectious diseases [3]. Mainly due to its high content of flavonoids, propolis has anti-inflammatory properties and promotes wound healing [4]. In dentistry, the positive effects of propolis-containing formulations have been shown in the treatment of peri-implant mucositis [5], gingivitis [6], periodontitis [7] and for caries prevention [8].

The most common oral diseases, caries, periodontal diseases and candidiasis result from an imbalance of the oral microbiota with the host response and/or ecological factors [9,10,11]. Caries is meanwhile seen as a bacterial-ecological approach, an endogenous infection leading to a disruption of biofilm homoeostasis; many bacteria become dominant besides the classic cariogenic bacteria, *Streptococcus mutans*, *Streptococcus sobrinus*, *Lactobacillus* and *Actinomyces* [12]. Periodontitis might be a bidirectional imbalance between the periodontal microbiome and the host response [13]. Bacteria highly present in the dysbiotic biofilm in periodontitis are, among others, *Porphyromonas gingivalis*, *Fretibacterium* ssp., *Treponema* ssp. and *Tannerella forsythia* [13]. In oral candidiasis, mostly *Candida albicans* is identified [14]. *C. albicans* has virulence factors to adhere to mucosa, and it synthesizes tissue-destroying enzymes, thus contributing to its invasiveness [15].

In a recent in vitro study, we compared the activity of three ethanolic extracts of propolis (EEP) originating from South America (red and green Brazilian propolis) and from Central Europe on microbial species associated with caries, periodontal disease, and with Candida infections [16]. All three EEPs were also highly active against oral microorganisms when organized in a biofilm. The Brazilian EEPs might better combat an already formed biofilm than the European EEPs. All EEPs inhibited the growth of oral microorganisms even in low concentrations, with the lowest values obtained by the green Brazilian propolis [16]. The source of the green Brazilian propolis is *Baccharis dracunculifolia* [17]. The chemical analysis made in the recent study showed 10 phenolic acids, 12 flavonoids and 2 prenylated derivatives of p-coumaric acid and acetophenone [16].

This follow-up in vitro study focused on the incorporation of the EEP of green Brazilian propolis (EEPg) in an oral health care product. Several series of experiments were scheduled. The first series dealt with a potential inflammation-modulating activity of propolis when a periodontal or Candida biofilm was exposed to monocytic cells. The research question to be answered was whether the EEPg reduces the inflammatory and promotes the anti-inflammatory response of the monocytic cells. The second series focused on the adhesion of *Porphyromonas gingivalis*, a major periodontopathogen, and of *Candida albicans* to gingival keratinocytes, which is an essential step in initiating disease development. Here, the research question to be answered was whether the EEPg reduces the potential of microorganisms to attach to epithelial cells. In the third series, EEP of green propolis was added to a commercially available toothpaste (TPcom). Enamel specimens were exposed to sucrose and a cariogenic biofilm for 14 d. The biofilm was removed daily by brushing with the toothpaste. The research question to be answered was whether EEPg may prevent substance microhardness loss. In the final, fourth series, an experimental sodium lauryl sulfate (SLS) free and erythritol (Ery) containing tooth paste formulation (Ery TP) was supplemented with EEPg. The research question to be answered was whether the EEPg in the formulation was as active as the EEPg itself regarding antimicrobial activity and preventing biofilm formation.

This study should give information on whether EEPg incorporated in a new toothpaste formulation may have the potential for beneficial effects in the prevention and/or therapy of caries, periodontal disease and candidiasis.

## 2. Results

As mentioned above, the experiments were made in different series; the first focused on the immunomodulating effect of EEPg on a monocytic cell line, the second on the influence of EEPg on the adhesion of microorganisms to gingival keratinocytes, the third on the anticariogenic effect when EEPg was added to a commercial toothpaste and the fourth on an experimental toothpaste formulation with EEPg.

### 2.1. First Series: Interaction with MONO-MAC-6 Cells

**Figure 1:**
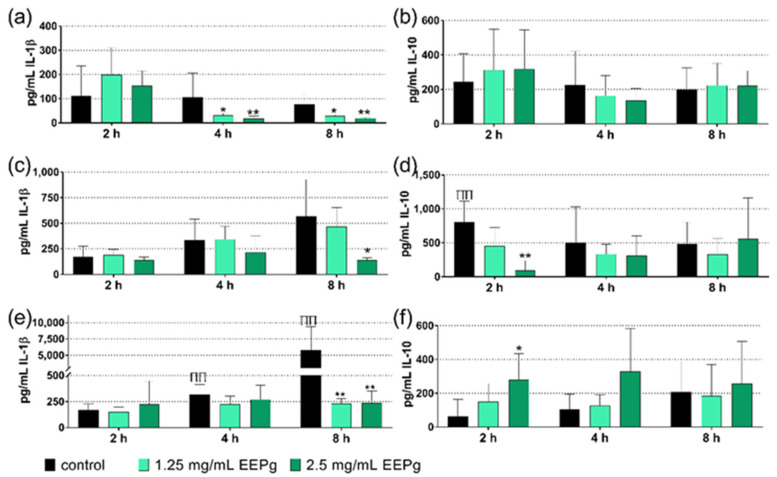
Released interleukin(IL)-1β (**a**,**c**,**e**) and IL-10 (**b**,**d**,**f**) from MONO-MAC-6 cells after being exposed to ethanolic extract of green propolis (EEPg) or ethanol without (**a**,**b**) and with a Candida biofilm (**c**,**d**) or with a periodontal biofilm (**e**,**f**) for 2, 4 and 8 h. Presented are mean and SD, */** *p* < 0.05/*p* < 0.01 vs. control of the respective time and assay. ^ΠΠ^ *p* < 0.01 vs. MONO-MAC-6 cells without biofilm at the respective time.

In this series, cells of a monocytic cell line of human origin (MONO-MAC-6 cells) were brought into contact with a Candida and periodontal biofilm and EEPg (1.25 mg/mL, 2.5 mg/mL) for 2 h, 4 h and 8 h. Thereafter, the released interleukin (IL)-1β (pro-inflammatory cytokine) and IL-10 (anti-inflammatory cytokine) were quantified. Also, cell controls without biofilm contact were analyzed (Figure 1a,b). Compared to control cells without biofilms (Figure 1a) or exposed to a Candida biofilm (Figure 1c), there was an increase in IL-1β when the MONO-MAC-6 cells were challenged with the periodontal biofilm for 4 h and 8 h (both *p* < 0.001) (Figure 1e). The release of IL-10 was higher when the Candida biofilm was in contact with the MONO-MAC-6 cells for 2 h (*p* < 0.001) (Figure 1d).

The addition of 1.25 mg/mL and 2.5 mg/mL of EEPg decreased the level of IL-1β released from MONO-MAC-6 cells after 4 h (*p* = 0.001, *p* = 0.015) and 8 h (*p* = 0.037, *p* = 0.001; Figure 1a); at 8 h also in the presence of the biofilms (Candida biofilm 2.5 mg/mL EEPg *p* = 0.049; periodontal biofilm 1.25 mg/mL EEPg *p* < 0.001, 2.5 mg/mL *p* = 0.003); Figure 1c,e. IL-10 was only affected after 2 h by 2.5 mg/mL EEPg in contact with biofilms (Figure 1d,f) but not without biofilm (Figure 1b). The high level of IL-10 in presence of the Candida biofilm was reduced (*p* < 0.001), whereas the level of IL-10 in contact with the periodontal biofilm was increased (*p* = 0.015).

**Figure 2:**
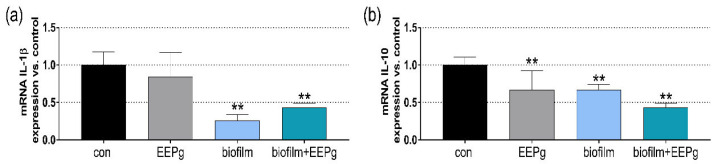
Monocytic (MONO-MAC-6-cells) mRNA expression of interleukin(IL)-1β (**a**) and IL-10 (**b**) after being exposed to 1.25 mg/mL ethanolic extract of green propolis (EEPg) and/or a periodontal biofilm for 30 min. Presented are mean and SD, ** *p* < 0.01 vs. control (The MONO-MAC-6 cells were tested for any cytotoxic reaction. Increased vitality of cells was found 2 h and 4 h after exposing them to 1.25 mg/mL or 2.5 mg/mL propolis; no difference existed after 8 h).

The IL-1β and IL-10 mRNA expression was measured after the MONO-MAC-6 cells had been challenged with 1.25 mg EEPg and/or periodontal biofilm for 30 min. EEPg alone only reduced IL-10 mRNA expression (*p* = 0.005). The periodontal biofilm suppressed both the IL-1β and IL-10 expressions (*p* < 0.001, *p* = 0.005). The biofilm together with EEPg reduced those of IL-1β and IL-10 vs. control (both *p* < 0.001; Figure 2a,b).

### 2.2. Second Series: Interaction with TIGK Cells

**Figure 3:**
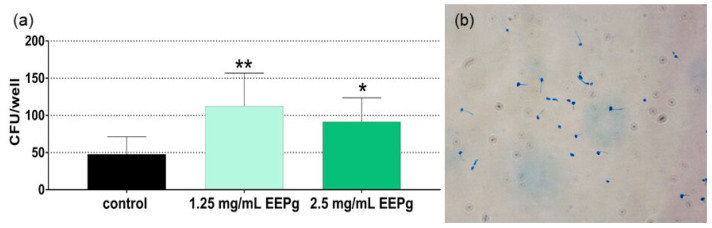
Adhering (incl. invaded) *Candida albicans* (colony forming units = cfu; mean and SD) to epithelial cells (**a**) in the presence of ethanolic extract of green propolis (EEPg) and formation of hyphae after being exposed to 2.5 mg/mL EEPg (**b**). Presented are mean and SD, */** *p* < 0.05/*p* < 0.01 vs. control.

In this series, the influence of EEPg (1.25 mg/mL, 2.5 mg/mL) on adhesion of microorganisms (*C. albicans*, *P. gingivalis*) to gingival keratinocytes (TIGK cells) after 1 h of joint incubation was analyzed. The two used concentrations of EEPg did not suppress the adhesion of *C. albicans* to the TIGK cells. In contrast, increased numbers were counted both after 1.25 mg/mL EEPg (*p* < 0.001) and after 2.5 mg EEPg (*p* = 0.011; Figure 3a).

Further, after the 2.5 mg EEPs exposure, the samples were stained with methylene blue. Microscopic photographs show a high percentage of the yeast with hyphae (Figure 3b).

As the results are not favorable related to a use of EEPg formulation in a Candida infection, *C. albicans* was not included in the further experiments.

**Figure 4:**
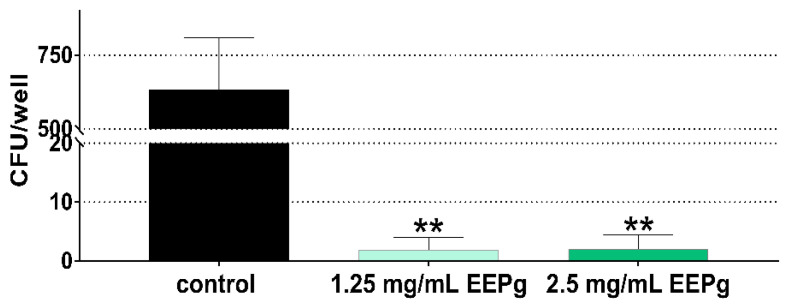
Adhering (incl. invaded) *Porphyromonas gingivalis* (colony forming units = cfu; mean and SD) to epithelial cells in the presence of ethanolic extract of green propolis (EEPg). Presented are mean and SD, ** *p* < 0.01 vs. control.

The results were different for *P. gingivalis*. Here, the number of adhered (incl. invaded) *P. gingivalis* was reduced to less than 0.5% in the samples with 1.25 mg/mL EEPg and 2.5 mg EEPg vs. controls (both *p* < 0.001; Figure 4).

A direct bactericidal effect of EEPg on *P. gingivalis* is obvious, as supernatant analysis found no viable bacteria after 2.5 mg/mL and a reduction to 20% after 1.25 mg/mL. However, a certain effect on cell vitality was found, with vitality of TIGK cells being reduced to about 40% already by 1.25 mg/mL of EEPg.

### 2.3. Third Series: Brushing, Biofilm Removal and Surface Microhardness Loss

**Figure 5:**
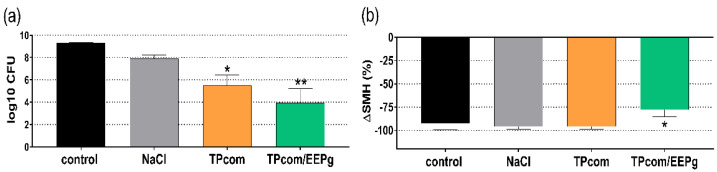
Bacterial counts (colony forming units = cfu; mean and SD) present in the biofilms (**a**), enamel surface microhardness (SMH) loss (**b**) of the samples in percent (mean and SD) after 14 days of exchange of media twice daily and daily brushing of the enamel specimens with slurries of a commercially available toothpaste (TPcom) without and with added 50 mg/mL ethanolic extract of green propolis (EEPg). Presented are mean and SD, */** *p* < 0.05/*p* < 0.01 vs. control.

In this series, EEPg was added to a commercial toothpaste (TPcom/EEPg). The cariogenic biofilm had been cultured repeatedly on enamel specimens for 14 d. Brushing with a toothpaste slurry or 0.9% *w*/*v* NaCl had been performed daily before the reformed biofilm and the surface microhardness loss was quantified. Frequent brushing with the TPcom without and with supplementation of EEPg resulted in less bacterial counts in the reformed biofilm at 14 d (TPcom: −3.81 log10, *p* = 0.019; TPcom/EEPg: −5.34 log10, *p* < 0.001; Figure 5a). The surface microhardness loss was high in all groups. It was in all groups above 90%, except in the TPcom/EEPg group, where it was only about 78%. The difference between the untreated control and the TPcom/EEPg group was statistically significant (*p* = 0.024; Figure 5b).

### 2.4. Fourth Series: Erythritol/Propolis Toothpaste

Experimental toothpaste formulations were created, Ery TP was supplemented with 25 mg/mL and 50 mg/mL EEPg. First, a potential growth inhibitory effect (minimal inhibitory concentration (MIC)) of Ery with and without Ery TP (as well as EEPg and TPcom as controls) was tested. Ery itself exerted some antibacterial activity. The MIC values against *A. naeslundii*, and *P. intermedia* were below 20 mg/mL. Similarly, the MIC values of the Ery TP were low against some strains. It became obvious that there was no growth inhibitory effect against the *S. mutans* strain. Compared to the Ery TP, the MICs of Ery TP with 25 mg/mL and 50 mg/mL EEPg were mostly lower, which suggests that the antibacterial effect of the EEPg was not blocked by the Ery TP. The results are presented in Table 1.

After MIC determinations, the effect of the experimental toothpaste formulations on biofilm formation was tested. The test substances (Ery TP with EEPg, Ery, EEPg in concentration of the toothpaste formulation; TPcom) were given to the surface before addition of microorganisms in nutrient broth in a ratio of 1:9. Three different biofilms were created: a two-species biofilm consisting of commensals, a cariogenic one and a periodontal one.

**Figure 6:**
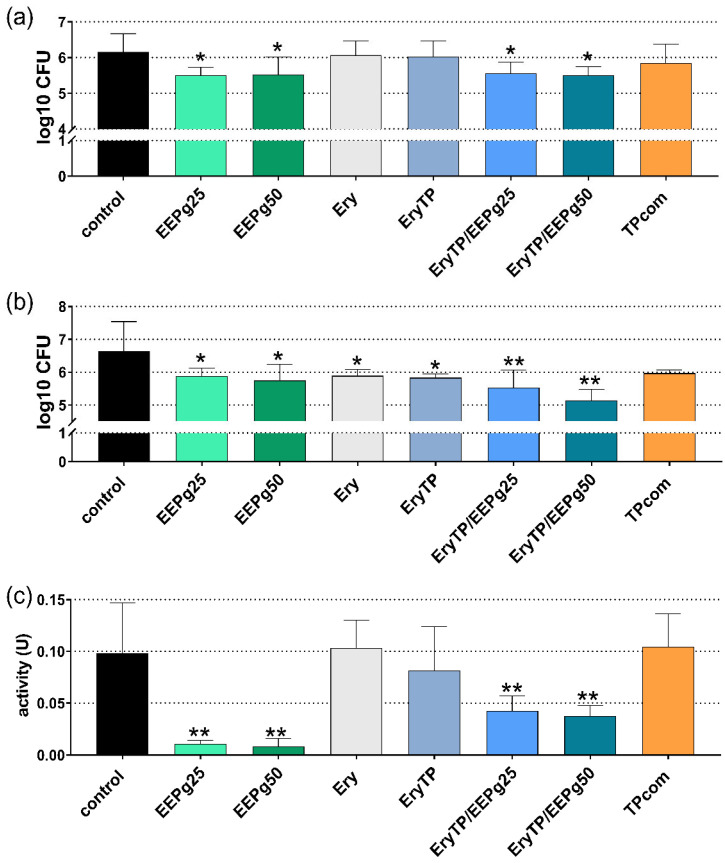
Activity of the ethanolic extracts of green propolis (EEPg, 25 mg/mL and 50 mg/mL), erythritol (Ery) and different toothpaste formulations (erythritol toothpaste (EryTP) without and with 25 mg/mL or 50 mg/mL EEPg, commercially available TP (TPcom)) on colony forming units (cfu); (**a**,**b**), and metabolic activity (**c**) in a formed two-species biofilm after 2 h (**a**) and 6 h (**b**,**c**). Presented are mean and SD, */** *p* < 0.05/*p* < 0.01 vs. control.

As the two-species biofilm represented the early colonizers, 2 h and 6 h of incubation were analyzed. At 2 h, the cfu counts were reduced after EEPg (25 mg/mL *p* = 0.032, 50 mg/mL *p* = 0.037) and after both Ery TPs with EEPg (25 mg/mL *p* = 0.048, 50 mg/mL *p* = 0.031; Figure 6a). The results were similar after 6 h (Figure 6b). Besides EEPg (25 mg/mL *p* = 0.030, 50 mg/mL *p* = 0.013) and Ery TP with EEPg (25 mg/mL *p* = 0.003, 50 mg/mL *p* < 0.001), the preapplication of Ery and Ery TP also resulted in less cfu counts vs. the control (*p* = 0.034; *p* = 0.022). At 6 h, the metabolic activity of the biofilm was found to be lower after EEPg (both *p* < 0.001) and after Ery TP with EEPg (25 mg/mL *p* = 0.005, 50 mg/mL *p* = 0.003; Figure 6c).

**Figure 7:**
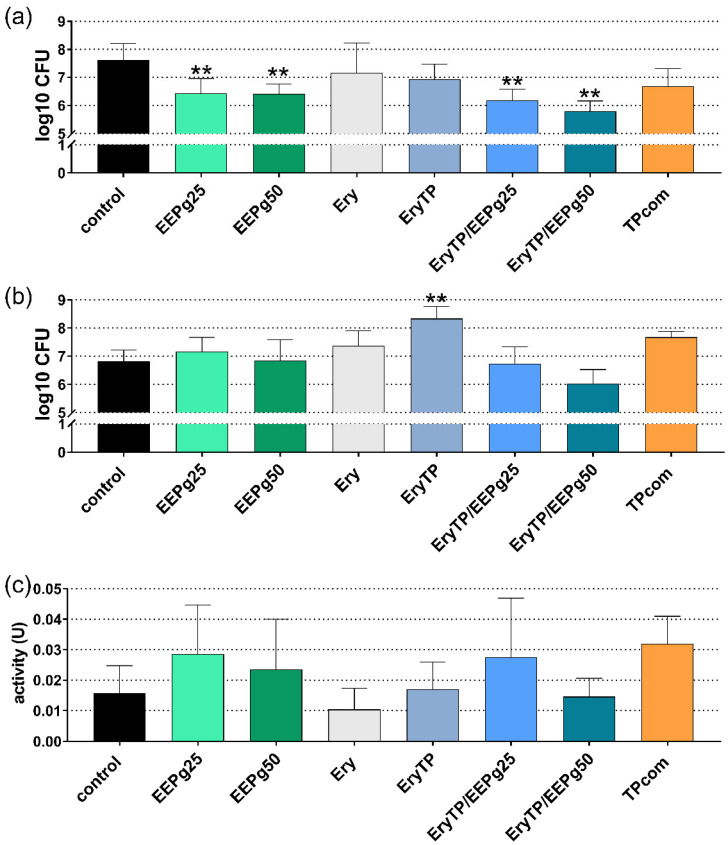
Activity of the ethanolic extracts of green propolis (EEPg, 25 mg/mL and 50 mg/mL), erythritol (Ery) and different toothpaste formulations (erythritol toothpaste (EryTP) without and with 25 mg/mL or 50 mg/mL EEPg, commercially available TP (TPcom)) on colony forming units (cfu); (**a**,**b**), and metabolic activity (**c**) in a formed cariogenic biofilm after 6 h (**a**) and 24 h (**b**,**c**). Presented are mean and SD, ** *p* < 0.01 vs. control.

Regarding the cariogenic biofilm, 6 h and 24 h of incubation were analyzed. At 6 h, the cfu counts were reduced after EEPg (25 mg/mL *p* = 0.004, 50 mg/mL *p* = 0.003) and after both Ery TP with EEPg (both *p* < 0.001; Figure 7a). The results were different after 24 h (Figure 7b). The cfu increased after Ery TP (*p* < 0.001); all other differences were not statistically significant vs. the control. Also, the metabolic activity of the 24-h biofilm did not show differences vs. the control; Figure 7c).

**Figure 8:**
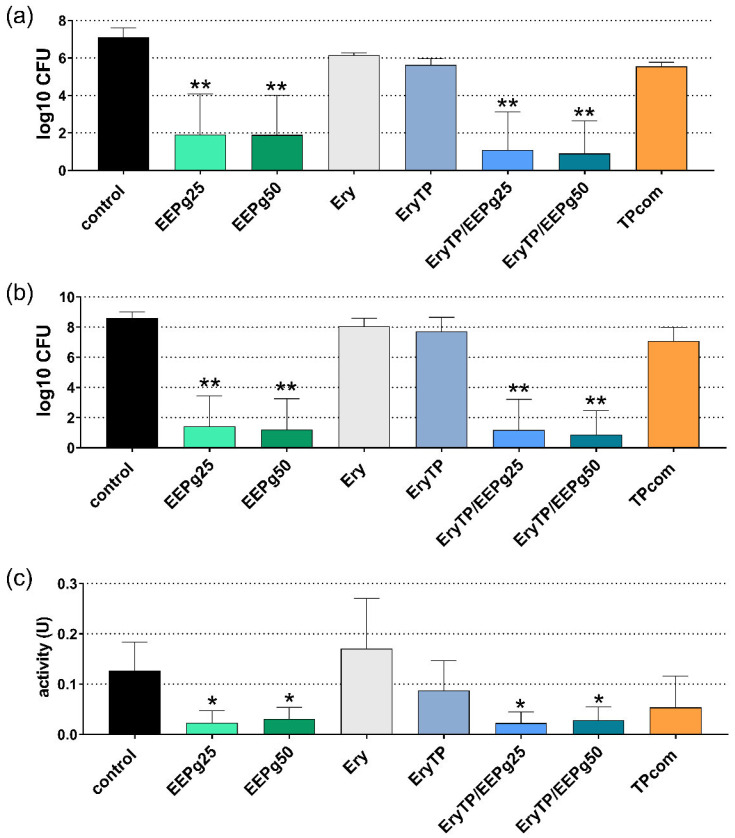
Activity of the ethanolic extracts of green propolis (EEPg, 25 mg/mL and 50 mg/mL), erythritol (Ery) and different toothpaste formulations (erythritol toothpaste (EryTP) without and with 25 mg/mL or 50 mg/mL EEPg, commercially available TP (TPcom) on colony forming units (cfu); (**a**,**b**), and metabolic activity (**c**) in a periodontal biofilm after 6 h (**a**) and 24 h (**b**,**c**). Presented are mean and SD, */** *p* < 0.05/*p* < 0.01 vs. control.

For the periodontal biofilm, 6 h and 24 h of incubation were analyzed. At 6 h, the cfu counts were reduced after 25 mg and 50 mg EEPg (both *p* < 0.001 vs. control) and after both Ery TP with EEPg (each *p* < 0.001; Figure 8a). The results were similar after 24 h (both EEPgs, both Ery TPs with EEPg *p* < 0.001, Figure 8b). At 24 h, the metabolic activity of the biofilm was found to be lower after EEPg (25 mg/mL *p* = 0.021, 50 mg/mL *p* = 0.044) and after Ery TP with EEPg (25 mg/mL *p* = 0.019, 50 mg/mL *p* = 0.036) vs. the control (Figure 8c).

## 3. Discussion

The invitro research was aimed to verify the potential of green propolis in ethanolic preparation as an ingredient in an oral health care product. Three different oral diseases were targeted (caries, periodontal disease and Candida infection). The results support an application in periodontal disease, and they also show, in part, the inhibition of the formation of a cariogenic biofilm and of a biofilm consisting of two early colonizers. However, an application in Candida infection is not supported by the present findings.

Candida infections and periodontal diseases are not only caused by biofilm itself, but they represent an interaction of the immune response to the microbes. In the innate immune response, macrophages are essential cells; they can be polarized into M1 and M2 [18]. M1 is known as a pro-inflammatory, microbiocidal stage and is characterized by the release of pro-inflammatory cytokines as IL-1β, whereas M2 stands for anti-inflammatory activities, having high phagocytotic activity and promoting wound healing; a predominant cytokine released by M2 phenotype is IL-10 [18]. In our experiments, a Candida biofilm initially increased the level of the anti-inflammatory IL-10 released by monocytic MONO-MAC-6 cells. This may confirm that *C. albicans* modulates the proteomic profile of macrophages into an M2 profile [19]. On the other hand, our periodontal biofilm induced the release of very high levels of IL-1β after 4 h and 8 h of incubation with the MONO-MAC-6 cells. At the mRNA expression level, a down-regulation was found; however, these experiments were made only after 30 min of incubation, which might be a limitation. Clinically high levels of IL-1β correlate to the severity of periodontitis and *P. gingivalis* counts [20]; periodontitis is characterized by an increase of the M1/M2 ratio [21].

Stimulating MONO-MAC-6 cells with EEPg was associated with an initial increase in cell vitality. We found decreased IL-1β levels when EEPg was added to the MONO-MAC cells. EEPg also inhibited the release of IL-1β when the MONO-MAC cells were challenged with the periodontal biofilm. A decrease in IL-1β expression by EEP was also reported by others. Using mouse macrophages challenged with LPS, an EEP of Iranian propolis decreased mRNA expression of IL-1β [22], and an EEP of Polish propolis suppressed both mRNA expression and secretion of IL-1β [23]. In contrast to IL-1β, the levels of IL-10 remained nearly unaffected. Only at 2 h, EEPg reduced the high IL-10 level induced by *C. albicans* and increased IL-10 in presence of a periodontal biofilm.

Our recent study showed that EEPg in a high concentration initially retarded the formation of a Candida biofilm. Further, it reduced the microbial counts and metabolic activity of a preformed Candida biofilm [16]. This suggests that EEPg may prevent candidiasis. In the present study, however, the increase in the number of adhered (incl. invasive) *C. albicans* to gingival keratinocytes was not in favor of EEPg. Microscopy showed, in addition, a high percent of hyphae morphology. The capability of forming pseudohyphae and hyphae promotes penetration into deeper tissues and represents a virulence factor [20]. Our result is in contrast to another study showing no change in the number of adhered *C. albicans* and an inhibitory effect on conversion from yeast to hyphae morphology [24]. Following our findings, it was decided not to focus further on Candida infections, although clinically, a propolis-containing gel was shown to prevent mucositis and Candida infection in patients undergoing radiation therapy in oral cavity [25].

Regarding caries prevention, EEPg might be helpful when added to a toothpaste. Thus, a commercial toothpaste was first supplemented with EEPg. Less bacteria in the reformed biofilm were counted, and less surface microhardness loss was measured after brushing enamel specimens with the toothpaste supplemented with EEPg once per day over 14 days. This confirms another in vitro study where an herbal-propolis toothpaste prevented mineral loss [26].

Finally, in our study, experimental toothpastes supplemented with two concentrations of EEPg were designed. The used EEPg concentrations in the toothpaste concentrations might be 10-fold higher than in the experimental settings when applying EEPg only. However, it can be assumed that the dilution of oral health care product occurs very fast by saliva or gingival crevicular fluid. A toothpaste formulation with no SLS but with 24% of erythritol was chosen. In vitro SLS-containing toothpastes were highly cytotoxic to gingival fibroblasts [27]; in-vivo nuclear morphological changes in buccal epithelial cells were observed [28]. The biocompatibility of this hydrophilic low abrasive toothpaste was shown to be very good [29]. The half lethal concentration (LC50) on a fibroblast cell line (L929) for the determination of tolerability for the oral mucosa resulted in up to 10-fold-higher LC50 values than in the other commercially available toothpastes [29].

Erythritol is a polyol of low molecular size and is produced by yeasts and lactic bacteria [30]. It is present in cheese and wine and is widely used as a low-caloric sweetener [30]. It has no effect on plasma glucose level and retards gastric emptying [31]. Erythritol is well tolerated and does not cause laxation, as is known to happen with other sugar replacements [32].

The effects of erythritol and of the erythritol toothpaste (without additives) on oral bacteria and biofilms were minor. Only the two-species biofilm was reduced after 6 h. This might be of relevance, as it reflects the effect on early colonizers. In another in vitro study, erythritol inhibited clearly in vitro the formation of a single-species biofilm of *S. gordonii* and of a dual species-biofilm when combined with *P. gingivalis* [33]. In our study, a more complex biofilm was not affected. This confirms that in saliva-biofilm models erythritol only slightly inhibited (about 0.5 log10 cfu) the cfu counts in biofilms after 9 d of erythritol exposure [34]. However, there, erythritol decreased the general proteolytic activity and the arginine-specific amidolytic activity [34], a finding which might be of relevance in a periodontal biofilm.

It is known that supplements in oral health care products may inhibit the activity of the antimicrobial compound; e.g., this in vitro study and clinical studies showed that for chlorhexidine-digluconate formulations containing anti-discoloration agents [35,36]. In the present analysis, the experimental toothpaste formulation retains to a high percent the antimicrobial activity of the EEPg. Regarding the microbial species, particularly good activity against bacteria associated with periodontal diseases was found. Commercial toothpaste comparisons found moderate antimicrobial activity of a propolis-containing toothpaste against *S. mutans* but not against *Enterococcus faecalis* and *Pseudomonas aeruginosa* [37].

In the final experiments, toothpaste was applied on a surface to verify a potential regrowth of biofilm after oral hygiene measures. Initially, but not after 24 h, a clear inhibition of biofilm formation was stated both by EEPg and by the toothpastes with EEPg. The initial retarding of biofilm formation might be of more clinical relevance than the result after 24 h, as tooth brushing twice per day is generally recommended [38]. Comparable studies cultured the biofilms for several days and applied in intervals the propolis toothbrush slurries [26,39]. In one study, propolis-containing toothpaste reduced more bacterial counts than one without active ingredients but less than one containing triclosan; the detailed analysis showed a suppression of *S. mutans* by the propolis toothpaste [26]. In the other study, bacterial counts in biofilms were similarly reduced, irrespective of whether the toothpaste contained propolis, chlorhexidine digluconate or no active antimicrobial [39].

The toothpaste with EEPg clearly retarded the formation of a biofilm associated with periodontal disease. Comparable in vitro studies on toothpastes are not available. In a mature multi-species biofilm, EEP of Brazilian red propolis reduced bacterial counts by about 0.4 log10 [40]. Our own experiments found a reduction of up to 2 log10 regarding a forming biofilm and the complete destruction of a preformed biofilm after 100 mg/mL EEPg [16].

A few toothpastes with propolis as an active ingredient are already commercially available. They have been tested in vitro [26,39] or in clinical trials [41,42]. A toothpaste with Polish propolis has been proven to be effective in the gingivitis prevention of patients with oral clefts [41]. A toothpaste preparation with EEP of green Brazilian propolis reduced biofilm and inflammation in patients with dental implants rehabilitation [42].

In our results on retarding biofilm formation, the EEPg containing toothpaste formulations were active, with no difference between the two used concentrations. This may imply that the lower concentration of 25 mg/mL might be sufficient, but this needs further evaluation.

In summary, erythritol toothpaste formulations with EEPg seem to have potential in prevention of caries, gingivitis and periodontitis and should be evaluated in further in vitro studies but also in clinical trials.

## 4. Materials and Methods

### 4.1. Propolis, Toothpastes

In the experiments, a 30% green propolis in ethanolic preparation (EEPg, Extrato de Própolis verde, APIS Flora, Sāo Paulo, Brasil) was used.

Erythritol (Ery) originated from Dr. Wittmann GmbH & Co. KG (Zwingenberg, Germany). The commercial toothpaste (TPcom) was Odol-med3^®^ Original (GlaxoSmithKline, Brentford, UK). According to the manufacturer’s information this toothpaste contains 1450 ppm sodium fluoride, aqua, hydrated silica, sorbitol, glycerin, sodium lauryl sulfate, xanthan gum, flavor, titanium dioxide, PEG-6, sodium saccharin, carrageenan, limonene, CI 73,360 and CI 74,160.

The experimental toothpaste (Ery TP; now on the market under the brand name AIRFLOW, Dr. Wittmann GmbH & Co. KG (Zwingenberg, Germany) was free of SLS. Instead, it contained the surfactant sodium C14-16 olefin sulfonate. It consisted of erythritol, xylitol and 1400 ppm sodium fluoride and had a slightly basic pH. The Ery TP was supplemented with 25 mg/mL and 50 mg/mL EEPg by intensive mixing immediately before the experiment.

### 4.2. Microorganisms

Overall, 16 microbial strains were included in the different experimental series. *Streptococcus gordonii* ATCC 10,558 and *Actinomyces naeslundii* ATCC 12,104 representing commensals were used. *S. mutans* ATCC 25,175, *S. sobrinus* ATCC 33,478 and *Lactobacillus acidophilus* ATCC 11,975 were representatives of cariogenic bacteria. *Fusobacterium nucleatum* ATCC 25,586, *Campylobacter rectus* ATCC 33,238, *Parvimonas micra* ATCC 33,270, *Eikenella corrodens* ATCC 23,834, *Prevotella intermedia* ATCC 2561, *Capnocytophaga gingivalis* ATCC 33,624, *Porphyromonas gingivalis* ATCC 33,277, *Tannerella forsythia* ATCC 43,037, *Filifactor alocis* ATCC 33,099 and *Treponema denticola* ATCC 35,405 cover the bacteria being associated with periodontal disease. The strain *Candida albicans* ATCC 76,615 was used in experiments, as it is related to Candida infections.

Besides *T. denticola* (modified mycoplasma broth (BD, Franklin Lake, NJ, USA)), strains were cultured on tryptic soy agar (TSA) plates (Oxoid, Basingstoke, UK; supplemented with 5% of sheep blood, in case of *T. forsythia* further with 10 mg/l N-acetyl muramic acid) at 37 °C in the respective atmosphere (streptococci, *A. naeslundii* with 10% CO_2_, *C. albicans* aerobically, others anaerobically).

### 4.3. Cells

In the assays, MONO-MAC-6-cells (DSMZ no. ACC 124) a monocytic cell line of human origin and telomerase-inactivated human gingival keratinocytes (TIGK) cells (ATCC-CRL-3397) were used. The cell cultivation media were RPMI 1640 medium supplemented with 10% fetal bovine serum (FBS; all Invitrogen; Carlsbad, CA, USA) for MONO-MAC-6 cells and Keratinocyte Growth Medium (KGM-Gold; Lonza, Basel, Switzerland) for TIGK cells. Before use in the experiments, MONO-MAC-6 cells were adjusted to 1.2 × 10^6^ cells/mL in RPMI 1640 medium with 0.5% FBS.

An influence on cell vitality by the experimental settings was screened by using MTT assay according to Mosmann [43].

### 4.4. Methods First Series: Interaction with MONO-MAC-6 Cells

A Candida and a periodontal biofilm were cultured in 96-well plates. All biofilms consisted of *S. gordonii* ATCC 10,558 and *A. naeslundii* ATCC 12,104, added by *C. albicans* ATCC 76,615 in case of the Candida biofilm and of 10 species associated with periodontal disease in case of the periodontal biofilm. Biofilms were created as published recently [16] and incubated for 48 h. At 48 h, the supernatant media was removed, and the biofilms were washed once very carefully. Then, 25 µL EEPg in a concentration of 12.5 mg/mL and 25 mg/mL EEP (equivalent to 3.75 mg/mL and 7.5 mg/mL green propolis) were added for one min before 225 µL MONO-MAC-6 cell suspension was pipetted per well. (Cell controls without biofilms were included.) That means that the final concentrations of EEPg were 1.25 mg/mL and 2.5 mg/mL. Thereafter, the 96-well plates were incubated for an additional 2 h, 4 h and 8 h. At these times, cell suspension was removed and centrifuged at 400 g before supernatants were taken and stored at −80 °C before assayed. Within 14 d thereafter, the levels of IL-1β and IL-10 were quantified using commercially available ELISA kits (R&D Systems, Minnesota, MN, USA) according to the manufacturer’s instruction. The detection levels were 1 pg/mL for both IL-1β and IL-10.

The protocol was accordingly for determining mRNA expression of IL-1β and IL-10. Here, only the periodontal biofilm was created and exposed to the final EEPg concentration of 1.25 mg/mL for 30 min. The total RNA was extracted using the innuPREP RNA Mini Kit 2.0 (Analytic Jena GmbH, Jena, Germany). Then, the GoScript™ Reverse Transcription System (Promega, Madison, WI, USA) was used to generate cDNA from RNA. The mRNA expression of IL-1β and IL-10 was quantified with qPCR (GoTaq^®^ qPCR Master Mix (Promega, Fitchburg, WI, USA)) and primers (IL-1β [44], IL-10 [45]. Gene expression was normalized by GAPDH (primer [46]) and analyzed by the 2^−ΔΔCT^ method.

### 4.5. Methods Second Series: Microbial Adhesion to TIGK Cells

TIGK cells were detached by trypsin/EDTA and adjusted to 1.2 × 10^5^/mL cell cultivation media. Each 0.25 mL was transferred to a well of a 24-well plate and cells were allowed to grow to confluency. Microorganisms were adjusted in PBS to OD 600 nm = 0.2 (about 1.6 × 10^8^ bacteria or 3 × 10^6^ yeasts per ml). EEPg was prepared in 10-fold concentration of the final one (final concentration 1.25 mg/mL and 2.5 mg/mL. Cell cultivation media was mixed with microbial suspension and EEPg solution in a ratio of 8:1:1. The confluent TIGK cells were washed two times before each 0.25 mL of the cell cultivation media-microbes-EEPg suspension was added. After short mixing, the plates were incubated with 5% CO_2_ for 1 h.

After washing, the cells were washed twice with PBS to remove non-adherent microbial cells and 1 mL ice-cold dH_2_O/well was added. The plates were left in place for 15 min. Then, the suspension was intensively mixed, and aliquots were plated on TSA agar plates. After incubation in the respective atmosphere, the numbers of colony forming units (cfu) were counted. This number stands for adherent including invasive microorganisms.

### 4.6. Methods Third Series: Substance Microhardness Loss

Enamel specimens were prepared as described recently [47]. In short, enamel specimens were prepared from extracted teeth (taking extracted teeth for research purpose was in accordance with the approved guidelines and regulations of the local ethical committee (KEK) for irreversibly anonymized samples), embedded in resin and polished. Specimens were placed in 24-well plates. A pellicle formation was simulated by adding 100 µL of a 0.27% pig gastric mucin solution for 1 h. Then the five microorganisms (*S. gordonii* ATCC 10,558, *A. naeslundii* ATCC 12,104 and cariogenic bacteria) were adjusted to McFarland 0.5 and mixed with same portions. The mixture was added to the culture media (brain heart infusion (BHI) broth, Oxoid) with 0.5% sucrose in a ratio of 1:50 and pipetted to the wells with the enamel specimens. At 6 h of incubation, the medium was carefully removed and replaced by a sucrose-free medium (BHI broth). After further incubation for about 18 h at 37 °C with 10% CO_2_, the specimens were placed in a sterile glass dish. Then, the enamel samples were brushed with 20 strokes for a total time of 10 s with a toothbrush and the commercially available toothpaste (TPcom), the TPcom supplemented with EEPg (final concentration 50 mg/mL). As a control, 0.9 *w*/*v* NaCl was used for brushing and another part of specimens was left without brushing. The toothbrush was a commercial one (Elmex Oeco-clic, GABA AG, Therwil, Switzerland) modified in order to allow the measurement of the brushing force [48], which was set to 1.5 ± 0.05 N. After brushing, the specimens were dipped into 0.9% *w*/*v* NaCl and placed in a new 24-well plate. This protocol with cariogenic biofilm formation, incubation in the two media and brushing were repeated 14 times, but finally, at 14 d, no brushing was performed.

At 14 d, specimens were dipped once into 0.9% *w*/*v* NaCl solution, and then biofilms were scraped from the enamel surface with a swab in 1 mL of 0.9% *w*/*v* NaCl. The total counts of cfu were determined. Further, at the beginning and at the end of the experiment, enamel surface microhardness (SMH) measurements were made using a Knoop diamond under a load of 50 gf and a dwell time of 10 s (UHL VMHT Microhardness Tester, UHL Technischer Mikroskopie, Aßlar, Germany) as described before [47]. The hardness value for the specimen was the mean value from six measurements. The difference of the measurements of the two time-points was calculated as the SMH loss in percent.

### 4.7. Methods Forth Series: Experimental Toothpaste

In these series, experimental toothpaste formulations were studied. First, the minimal inhibitory concentration (MIC) values of the toothpaste formulations and related ingredients was determined against seven oral microorganisms: *S. gordonii* ATCC 10,558, *A. naeslundii* ATCC 12,104, *S. mutans* ATCC 25,175, *F. nucleatum* ATCC 25,586, *P. micra* ATCC 33,270, *P. intermedia* ATCC 2561 and *P. gingivalis* ATCC 33,277. Of the test substances, a two-fold dilution series was prepared. A defined inoculum of the microorganisms was added to a two-fold concentrated broth, which was mixed in a ratio of 1:1 with the test substances in different concentrations. As the test substances caused turbidity of the media, after an incubation time of 42 h (18 h aerobes) the growth of microbes was analyzed by subcultivation. MIC represented the lowest concentration with clear growth inhibition. These experiments were made in independent replicates.

Further, the inhibition of biofilm formation was tested. Wells of a 96-well plate were covered with 25 µL of test substances (25 mg/mL EEPg, 50 mg/mL EEPg, 24% Ery, Ery Tp, Ery TP with 25 mg/mL EEPg or 50 mg/mL EEP) for 1 min before biofilm formation was made as described before [16]. In short, after applying a protein solution, the bacterial suspension mixed with nutrient broth was added. Three types of biofilms were used: a two species biofilm consisting of *S. gordonii* ATCC 10,558 and *A. naeslundii* ATCC 12,104, a five-species cariogenic biofilm and a 12-species periodontal biofilm. In case of the two-species biofilm, the analysis was made after 2 h and 6 h; in case of the other biofilms, it was made after 6 h and 24 h of development of biofilm. The biofilms were incubated at 37 °C with 10% CO_2_ or in an anaerobic atmosphere (periodontal biofilm). At the respective times, biofilms were removed from the surface to assess the total cfu counts and to determine the metabolic activity of the biofilm suspension using Alamar blue as a redox indicator [49].

### 4.8. Statistical Analysis

Except for the MIC determinations of the toothpastes and related ingredients, all other experiments were made in at least two independent experiments each with independent quadruplicates. In the graphs, the data are presented as mean and standard deviation. In case of microbial counts, log10 values were generated and used in the analysis.

Statistical analysis was made using a one-way analysis of variance (ANOVA) with post-hoc Bonferroni with the help of SPSS 28.0 (IBM, Chicago, IL, USA) software. A *p*-value of 0.05 was considered to be statistically significant.

## Figures and Tables

**Table 1:** Minimal inhibitory concentrations (mg/mL) of erythritol (Ery), an Ery toothpaste (Ery TP, containing 15% erythritol), ethanolic extract of green propolis (EEPg, containing 30% green propolis), Ery TP supplemented with EEPg and a commercially available toothpaste (TPcom).

Strain	Ery	Ery TP	EEPg	Ery TP + 50 mg/mL EEPg	Ery TP + 25 mg/mL EEPg	TPcom
*S. gordonii* ATCC 10,558	125	62.5	<0.1	<0.1	31.3	31.3
*A. naeslundii* ATCC 12,104	15.6	15.6	<0.1	<0.1	7.8	15.6
*S. mutans* ATCC 25,175	500	>500	0.2	0.2	31.3	31.3
*F. nucleatum* ATCC 25,586	125	250	0.2	0.2	7.8	7.8
*P. micra* ATCC 33,270	500	62.5	<0.1	<0.1	7.8	7.8
*P. intermedia* ATCC 25,611	15.6	7.8	<0.1	<0.1	7.8	7.8
*P. gingivalis* ATCC 33,277	62.5	7.8	0.2	0.2	2	2

## Data Availability

The data presented in this study are available on request from the corresponding author.
